# Disparities in practice: implementation of health promotion policies in 2883 Hungarian schools with a focus on small-sized institutions

**DOI:** 10.1093/eurpub/ckag143

**Published:** 2026-08-13

**Authors:** Zsuzsa Rákosy, Dorottya Árva, Viktória Anna Kovács

**Affiliations:** Department of Public Health Medicine, Medical School, University of Pécs, Pécs, Hungary; Directorate of Prevention, Bethesda Children’s Hospital, Budapest, Hungary; MTA-PTE Innovative Health Pedagogy Research Group, Pécs, Hungary; MTA-PTE Innovative Health Pedagogy Research Group, Pécs, Hungary; Institute of Preventive Medicine and Public Health, Faculty of Medicine, Semmelweis University, Budapest, Hungary; Directorate of Prevention, Bethesda Children’s Hospital, Budapest, Hungary; Institute of Health Sciences, Károli Gáspár University of the Reformed Church, Budapest, Hungary

## Abstract

While Holistic Health Promotion is mandated in Hungary, little is known about its school-level implementation, especially in small schools. We analyzed national data of 2883 elementary schools, and (i) compared the implementation between small (10–99 students) and not-small schools, and (ii) conducted county-level socioeconomic comparisons within the subsample of small schools. Health promotion activities were implemented inconsistently across schools. Small schools (*N *= 320) reported fewer structured working groups (9.7%) and lower engagement of school health staff in program development (doctors: 3.1%, nurses: 6.9%, psychologists: 0.9%). Participation in national food schemes was markedly higher in low socioeconomic status (SES) areas than in high SES small schools (Milk Scheme: 83.5% vs. 46.5; Fruit & Vegs Scheme: 86.9% vs. 67.3%). Menu planning was aligned with the Public Catering Act in two-thirds of all schools (68.9%), which falls short of full adherence. Coverage of nutrition education was high (95.5%). Experiential learning tools like school gardens (31.7%) and educational kitchens (18.8%) were predominantly found in high SES context. After-school physical activity opportunities were significantly limited in small schools. The availability of bullying (32.2%) and digital addictions (52.2%) prevention measures was limited in low SES context. Parental health literacy programs showed moderate integration across all groups (average score: 6 out of 10). This study provides the first nationwide school-level evidence on variation in the implementation of health promotion policies. Findings highlight modest differences by size and SES, and underscore the need for targeted actions to support small and disadvantaged schools.

## Introduction

Despite ongoing improvements, health outcomes of the Hungarian population remain poor, placing Hungary among the countries with the worst health status in the region [1]. These adverse outcomes are largely driven by behavioral risk factors such as unhealthy diets and insufficient physical activity [1], which are already prevalent among school-aged children [2]. According to the most recent Health Behaviour in School-aged Children study, 29% of Hungarian girls and 22% of boys are highly inactive, daily fruit consumption varies between 23% and 47% [2], and the prevalence of overweight (including obesity) reaches 34% among boys and 21% among girls [2].

In this context, schools represent a strategic setting for health promotion [3]. Accordingly, the Health Promoting Schools (HPS) approach has emerged as a whole-school framework integrating health into all aspects of school life to promote, protect, and nurture students’ physical, mental, and social well-being while also improving life skills and educational outcomes [3].

Hungary represents a particularly relevant context for examining school-based health promotion. Compulsory education until the age of 16 years ensures long-term, nearly universal access to children. Furthermore, the centrally governed education system facilitates coordinated nationwide implementation of school health policies. This policy commitment is operationalized through the Hungarian model of Holistic Health Promotion (HHP), which has been mandatory for all public education institutions since 2012.

The HHP model is closely aligned with HPS principles [4, 5], but differs in governance and implementation. While HPS primarily encourages voluntary adoption, HHP is mandated through national legislation [6]. Consequently, the Hungarian context has shifted from motivating schools to engage in health promotion toward supporting the implementation of legally prescribed activities, and challenges relate primarily to implementation quality [7]. In this process, schools are supported by guidelines and educational materials, and health promotion may involve not only teachers but also school psychologists, nurses, and doctors, although availability of these professionals depends strongly on school size. External experts may contribute only if accredited by the National Centre for Public Health and Pharmacy.

Similar to HPS, HHP adopts a whole-school approach by embedding health promotion into school life, educational processes, environments, and partnerships with families and health professionals [4, 5]. However, while HPS frameworks typically allow schools to determine priority topics locally, Hungarian regulations prescribe continuous, age-appropriate, and measurable activities across four core domains: healthy diet, daily physical activity, health literacy, and mental health, while also addressing addiction prevention, violence, and early school leaving.

Evidence shows that the HPS approach, characterized by collaboratively addressing multiple domains through a combination of strategies is effective [8, 9]. Their sustainability depends on policy support, institutionalization, and alignment with the local context [10]; yet, implementation is often uneven and project-based, depending heavily on local engagement [11]. Supportive school leadership, staff ownership, organizational readiness for change, local champions, trainings, sufficient resources are among the key facilitators [12]. Schools are also more likely to adopt and sustain HPS practices when they perceive clear educational benefits [11]. Conversely, limited time and confidence, heavy teacher workload, and unclear responsibilities are commonly reported as main implementation barriers [12].

Despite the long-standing policy framework, school-level data remain scarce. Hungary has participated in monitoring studies coordinated by the Schools for Health in Europe (SHE) Network Foundation; however, these reports provided mainly aggregated, indirect insights from the perspective of the national coordinator [13–15]. These evaluations suggest a top-down, policy-driven implementation model in Hungary, with health promotion activities largely integrated into existing interventions and often delivered through passive learning methods across subjects. Parental involvement is a common strategy, while barriers such as teacher workload and facilitators like legal mandates have been identified [7]. Also, the practical integration of HHP into core educational documents, such as curricula, pedagogical programs and implementation guidelines, has remained limited [16]. Furthermore, neither the National Core Curriculum nor the associated framework curricula define grade-specific learning outcomes for health promotion.

School size and socioeconomic context may further influence implementation. Small schools are often characterized by closer social relationships, stronger cohesion, and safer environments [17–19], but may simultaneously face resource limitations and reduced access to specialized services, potentially constraining health promotion activities [20, 21]. Similarly, extensive evidence demonstrates that lower socioeconomic status (SES) is associated with higher exposure to behavioral risk factors and more limited access to health-promoting resources [22–25]. These inequalities extend to schools, where disadvantaged settings may face limitations in staffing, facilities, and parental engagement, thereby restricting implementation capacity [26]. We therefore hypothesized that health promotion activities would be implemented more frequently in economically resourceful areas, even among small schools.

Given the lack of nationwide school-level assessment of HHP implementation, this study aimed to evaluate HHP implementation across a large sample of Hungarian schools within the legally mandated policy context. The study examines the status of implementation 8 years after its introduction, and explores its association with school size and SES context. Two objectives were defined: first, to compare implementation between small and not-small schools nationwide; second, to examine how county-level socioeconomic disparities shape implementation within the subsample of small schools. Out of the four core HHP domains, the current work focuses on healthy diets and physical activity. Results on mental health and addiction prevention were published elsewhere [27].

## Methods

We used data from a quantitative survey that assessed the status of health promotion activities in October 2019. The study was approved by the Ethical Review Board of the National Korányi Institute of Pulmonology, Budapest, Hungary, and conducted in accordance with local legislation and institutional requirements.

### Participants

The target population comprised Hungarian elementary schools, which provide primary and lower secondary education within a single institutional structure from age 6 to 14 years. Detailed information on school selection and survey tools can be found in Árva et al. [27]. Briefly, principals of all Hungarian elementary schools (*N* = 3601) were contacted by email by the ministry responsible for health and education. Participation was voluntary and anonymous, and all schools with complete survey records were included.

The aim was not to obtain a statistically representative sample, but to reach the full national school population. Because invitations were distributed centrally, broad national coverage was expected. To assess geographic distribution, county-level proportions in the sample were compared with the national distribution of schools [28]. This comparison was intended to evaluate territorial coverage rather than formal representativeness.

### Survey instrument

The online questionnaire contained 32 items, of which 15 were analyzed in the present study. Items addressed key aspects of HHP implementation related to healthy diet and physical activity, including school characteristics, stakeholder involvement, and implementation practices. The questionnaire was developed according to the official HHP policy domains and conceptually aligned with the 2021 WHO Global Standards for HPS [3]. Survey items were retrospectively mapped to HPS domains covering school policies and resources, governance and leadership, community partnerships, curriculum, social-emotional and physical environment, and school health services (Table 1). Most variables were dichotomous (yes/no), while one item assessing support for parental health literacy used a Likert-type scale (0–10).

**Table 1.** Links between questionnaire items and the 2021 WHO Health Promoting Schools Global Standards [3]^a^

WHO HPS global standard	Description of the standard	Corresponding survey items	Operational focus in this study
Standard 2—School policies and resources	Institutional commitment, partnership engagement, adequate resources, planning and monitoring processes	Q10–Q11 (participation in School Milk and Fruit and Vegetable Schemes); Q12-1 (compliance with Public Catering Act); Q12-7 (ensuring special dietary needs)	Presence of formal structures and policy-driven nutrition support
Standard 3—School governance and leadership	Leadership team, participatory leadership, leaders’ training, performance monitoring	Q5 (health promotion working group); Q6-1–6 (involvement of teachers, school doctor, nurse, psychologist, parents, students in HP program development)	Leadership, inclusive planning and shared responsibility
Standard 4—School and community partnerships	Formal and informal collaboration with families and local community actors	Q6-5 (parental involvement); Q12-6 (use of local products in public catering); Q30 (programs to increase parental health literacy and healthy lifestyle choices)	School–family partnership and outreach to local producers
Standard 5—School curriculum	Integration of health into teaching and learning, adequate staff training	Q13-1 (nutrition education); Q13-7 (conscious shopping education)	Health education and skills development
Standard 6—School social-emotional environment	Safe, supportive, and inclusive climate	Q21-1 (Rhythmic movements to music, e.g. dance); Q21-2 (stress-relief techniques); Q24-5 (digital addiction prevention); Q24-6 (prevention of body image perception disorders); Q24-7 (bullying prevention)	Psychosocial wellbeing and supportive school climate through preventive and educational activities
Standard 7—School physical environment	Infrastructure and spaces are safe, secure, healthy and inclusive	Q13-2 (healthy menu in public catering); Q13-4 (water fountains); Q13-5 (school garden); Q13-6 (educational kitchen); Q22-1–4 (access to sports facilities and activities)	Physical and infrastructural environment enabling healthy behaviors
Standard 8—School health services	Universal access to comprehensive health services	Q6-2–4 (school doctor, nurse, psychologist involvement); Q13-8 (school health day)	Involvement of school health professionals in health promotion activities

a The questionnaire was primarily designed to assess implementation of Hungary’s legally mandated holistic school health promotion policy. Survey items were retrospectively mapped to the WHO Health Promoting Schools Global Standards to demonstrate conceptual alignment with the whole-school approach.

HPS, Health Promoting Schools; Q, question; WHO, World Health Organization.

### Data analysis

Descriptive statistics summarized school characteristics. Continuous variables were reported as medians with 95% confidence intervals (CI), while categorical variables were presented as frequencies and percentages. School size was categorized as small (10–99 students), medium (100–500), or large (501+ students) based on the Hungarian public education indicator system [29]. Counties were grouped into SES terciles (low, medium, high) using composite economic indicators including gross domestic product per capita, average earnings, and labor market activity [30].

Pearson’s chi-square tests were applied in two analytical steps. First, implementation differences were examined by school size, comparing small schools with not-small schools (medium and large combined). Second, SES-related differences were examined within the subsample of small schools. Cramer’s *V* was calculated to assess effect size. Statistical significance was set at *P* < .05. Analyses were performed using R software.

To provide an aggregated view of implementation aligned with the HPS Global Standards, exploratory standard-level indicators were constructed based on the mapping shown in Table 1. For each school, the proportion of implemented items within a standard was calculated as the number of “yes” responses divided by the total number of mapped items. Likert-type responses were recoded as “implemented” for scores of 8–10. School-level proportions were averaged across groups and expressed as percentages. These indicators reflect the density of implementation within each standard. Given their exploratory nature and equal weighting of heterogeneous items, they were treated as descriptive summaries without formal statistical testing.

## Results

### Sample characteristics

A total of 2892 schools completed the questionnaire; 9 (0.3%) reporting fewer than 10 students were excluded, resulting in a final sample of 2883 schools. The county-level distribution of responding schools closely reflected the national distribution, indicating good geographic coverage and reducing the likelihood of major regional bias (Supplementary Figure 1). Sample characteristics, including *P* values and effect sizes for group comparisons, are presented in Table 2. Most schools were medium-sized, while small schools represented 11.1% of the sample. SES analyses in subsequent tables were conducted only within the subsample of small schools, which was relatively evenly distributed across SES categories. Public schools accounted for most responses, followed by church-maintained institutions, whereas private schools were more common among small schools, particularly in high SES areas, where they represented 28.7% of schools compared with 4.4% in low SES areas (*P* < .001).

**Table 2.** Sample characteristics and health promotion governance by school size (full sample) and by SES within small schools in Hungary (*N *= 2883)^a^

	All schools (*N *= 2883)	Not-small schools^b^ (*N = *2563)	Small schools^b^ (*N *= 320)	*P* (size)^c^ (effect size) (*N *= 2883)	Low SES small schools (*N *= 115)	High SES small schools (*N *= 101)	*P* (SES)^c^ (effect size) (*N *= 320)
**Size^b^**							
Small (10–99 students)	320 (11.1)						
Medium (100–500 students)	1919 (66.6)						
Large (501+ students)	644 (22.3)						
**SES status^d^**							
High	1261 (43.7)	1160 (45.3)	101 (31.6)				
Medium	767 (26.6)	664 (25.9)	104 (32.5)				
Low	855 (29.7)	739 (28.8)	115 (35.9)				
**Maintainer**							
Public	2326 (80.7)	2089 (81.5)	237 (74.1)	**<.001** (0.13)	98 (85.2)	61 (60.4)	**<.001** (0.33)
Church	403 (13.9)	372 (14.5)	31 (9.7)		12 (10.4)	11 (10.9)	
Private	154 (5.3)	102 (4.0)	52 (16.3)		5 (4.4)	29 (28.7)	
**Health promotion working group**							
Yes	403 (14.0)	372 (14.5)	31 (9.7)	**.022** (0.04)	12 (10.4)	9 (8.9)	.061 (0.11)
No	2470 (85.9)	2181 (85.1)	289 (90.3)		103 (89.6)	92 (91.1)	
Missing data	10 (0.4)	10 (0.4)	0		0	0	
**Involvement in health program development**							
Teachers	413 (14.3)	381 (14.9)	32 (10.0)	**.024** (0.04)	12 (10.4)	9 (8.9)	.907 (0.02)
School doctor	187 (6.5)	177 (6.9)	10 (3.1)	**.014** (0.05)	3 (2.6)	2 (1.9)	.470 (0.05)
School nurse	336 (11.7)	314 (12.3)	22 (6.9)	**.006** (0.04)	8 (6.9)	7 (6.9)	.913 (0.01)
School psychologist	124 (4.3)	121 (4.7)	3 (0.9)	**.003** (0.06)	0 (0.0)	2 (1.9)	.321 (0.09)
Parents committee	144 (4.9)	130 (5.1)	14 (4.4)	.686 (0.01)	7 (6.1)	2 (1.9)	.327 (0.09)
Students	109 (3.8)	99 (3.9)	10 (3.1)	.619 (0.01)	3 (2.6)	2 (1.9)	.470 (0.05)

a Data are *N*(%). Missing values are only reported where missing data were present. Effect sizes (Cramér’s *V*) are reported in parentheses next to the corresponding *P* values.

b Cut-off points were selected in accordance with the official thresholds used in the Hungarian public education indicator system (*Hungarian Academy of Sciences, 2017*).

c Pearson’s chi-square tests were conducted in two analytical steps: (i) by school size, comparing small schools (10–99 students) with not-small schools (≥100 students, combining medium and large schools); and (ii) within the subsample of small schools only, comparing SES terciles (low vs. medium vs. high). SES, socioeconomic status.

d Counties in which schools were operated were grouped into three SES terciles (low, medium, high) based on composite economic indicators (*Economx, 2022*).

Bold values indicate statistically significant differences (*p* < 0.05).

### School governance and planning

Only 14% of all schools had a dedicated health promotion working group, with an even lower proportion among small schools (9.7%), though effect sizes were small (Table 2). The involvement of stakeholders in health program planning was low across all groups, with modest variation. Teachers were the most involved group, but still only in 14.3% of all schools, dropping to 10.0% in small schools. Overall, students showed the lowest involvement level (3.8%) with the smallest rate in high SES small schools (1.9%). School psychologists were involved in less than 1% of small schools and not at all in low SES small schools.

### Food and nutrition

Small schools were participating more frequently in national food schemes than medium and large schools (*P* < .05), though effect sizes were small. Additionally, we observed a difference in the participation in School Milk Scheme between low and high SES small schools (*P* < .001, *V* = 0.32) with medium effect size (Table 3). Implementation of the Fruit and Vegetable Scheme reached 87.0% in low SES small schools vs. 67.3% of high SES small schools (*P* < .001, *V* = 0.22). Approximately 70% of all schools reported compliance with the Public Catering Act, with no variation across SES or school size. High SES small schools were more likely to consider parents’ preferences during menu planning than low SES small schools (13.9% vs. 2.6%, *P* < .05, *V* = 0.18). Local products were used more often in low SES small schools (16.5%) than in high SES small schools (4.9%; *P* < .001, *V* = 0.19). Only 23.5% of low SES small schools reported meeting the needs of children with special dietary requirements, compared to 47.5% in high SES small schools (*P* < .001, *V* = 0.21). Regarding school-based strategies to support healthy eating, nutrition education was widespread overall (95.5%). Teachers served as role models more frequently in small schools than in the larger ones (51.9% vs. 42.2%, *P* = .002); however, effect size was very small. Access to water fountains was lower in high SES small schools than in low SES areas with no statistically significant difference (26.7% vs. 38.4%; *P* = .064). Experiential food education (e.g. school gardens and educational kitchens) was more common in high SES small schools (31.7% and 18.8%, respectively) than in low SES small schools (17.4% and 8.7%; *P* < .05), but effect sizes were small. School health days were organized less frequently in small schools compared to larger ones (68.4% vs. 76.2%; *P* = .003) with a small effect size.

**Table 3.** Implementation of diet, physical activity, and obesity-related health promotion policies by school size (full sample) and by SES within small schools in Hungary (*N *= 2883)^a^

	All schools (*N *= 2883)	Not-small schools (*N = *2563)	Small schools^b^ (*N *= 320)	*P*(size)^c^ (effect size) (*N *= 2883)	Low SES small schools^d^ (*N *= 115)	High SES small schools^d^ (*N *= 101)	*P*(SES)^c^ (effect size) (*N *= 320)
**Participation in National Food Schemes**							
School milk program	1572 (54.5)	1357 (52.9)	215 (67.2)	**<.001** (0.13)	96 (83.5)	47 (46.5)	**<.001** (0.32)
School fruit and vegs program	2164 (75.1)	1908 (74.4)	256 (80.0)	**.036** (0.04)	100 (86.9)	68 (67.3)	**<.001** (0.22)
**Menu planning considerations**							
Public catering act	1986 (68.9)	1760 (68.7)	226 (70.6)	.517 (0.01)	85 (73.9)	67 (66.3)	.470 (0.07)
Students’ preferences	682 (23.7)	601 (23.4)	81 (25.3)	.503 (0.02)	23 (20.0)	33 (32.7)	.095 (0.12)
Parents’ preferences	304 (10.5)	281 (11)	23 (7.2)	**.048** (0.04)	3 (2.6)	14 (13.9)	**.005** (0.18)
Teachers’ preferences	234 (8.1)	217 (8.5)	17 (5.3)	.066 (0.03)	3 (2.6)	8 (7.9)	.214 (0.10)
Use of local products	267 (9.3)	238 (9.3)	29 (9.1)	.978 (0.00)	19 (16.5)	5 (4.9)	**.002** (0.19)
Special dietary needs	1388 (48.1)	1273 (49.7)	115 (35.9)	**<.001** (0.12)	27 (23.5)	48 (47.5)	**<.001** (0.21)
**Strategies to support healthy eating**							
Nutrition education	2754 (95.5)	2454 (95.7)	300 (93.8)	.137 (0.03)	110 (95.7)	91 (90.1)	.185 (0.10)
Healthy menus in public catering	978 (33.9)	858 (33.5)	120 (37.5)	.170 (0.02)	46 (40.0)	37 (36.6)	.778 (0.04)
Teachers as role models	1252 (43.4)	1086 (42.4)	166 (51.9)	**.002** (0.06)	57 (49.6)	50 (49.5)	.483 (0.07)
Water fountains	1161 (40.3)	1050 (41)	111 (34.7)	**.036** (0.04)	40 (34.8)	27 (26.7)	.064 (0.13)
School garden	580 (20.1)	500 (19.5)	80 (25.0)	**.025** (0.05)	20 (17.4)	32 (31.7)	**.046** (0.14)
Educational kitchen	568 (19.7)	514 (20.1)	54 (16.9)	.203 (0.03)	10 (8.7)	19 (18.8)	**.008** (0.17)
Education on healthy shopping	1390 (48.2)	1251 (48.8)	139 (43.4)	.079 (0.03)	53 (46.1)	47 (46.6)	.330 (0.08)
School health day	2173 (75.4)	1954 (76.2)	219 (68.4)	**.003** (0.06)	83 (72.2)	64 (63.4)	.372 (0.08)
**Features of daily PE lessons**							
Rhythmic movements	1637 (56.8)	1475 (57.5)	162 (50.6)	**.022** (0.04)	56 (48.7)	47 (46.5)	.301 (0.09)
Stress-relief techniques	2007 (69.6)	1777 (69.3)	230 (71.9)	.385 (0.02)	81 (70.4)	74 (73.3)	.897 (0.03)
None	366 (12.7)	320 (12.5)	46 (14.4)	.385 (0.02)	18 (15.7)	14 (13.9)	.885 (0.03)
**Access to after school sports**							
Free access to school facilities	2000 (69.4)	1821 (71)	179 (55.9)	**<.001** (0.13)	67 (58.3)	55 (54.5)	.820 (0.04)
Free/supported access to external facilities	1041 (36.1)	951 (37.1)	90 (28.1)	**.002** (0.06)	28 (24.4)	28 (27.7)	.388 (0.08)
Free in-school sport activities	2308 (80.1)	2091 (81.6)	217 (67.8)	**<.001** (0.12)	75 (65.2)	62 (61.4)	**.045** (0.14)
Fee-based in-school sport activities	531 (18.4)	511 (19.9)	20 (6.3)	**<.001** (0.13)	6 (5.2)	9 (8.9)	.407 (0.07)
None	105 (3.6)	72 (2.8)	33 (10.3)	**<.001** (0.12)	11 (9.6)	14 (13.9)	.330 (0.08)
**Obesity-related prevention programs**							
Internet and digital addictions	1758 (60.7)	1588 (62)	170 (53.1)	**.003** (0.06)	60 (52.2)	59 (58.4)	.392 (0.08)
Body image perception disorders	608 (21.1)	550 (21.5)	58 (18.1)	.192 (0.03)	15 (13.0)	22 (21.8)	.201 (0.10)
Bullying and school violence	1472 (51.1)	1353 (52.8)	119 (37.2)	**<.001** (0.12)	37 (32.2)	41 (40.6)	.375 (0.08)

a Data are *N*(%). Missing values are only reported where missing data were present. Effect sizes (Cramér’s *V*) are reported in parentheses next to the corresponding *P* values.

b Cut-off points were selected in accordance with the official thresholds used in the Hungarian public education indicator system (*Hungarian Academy of Sciences, 2017*).

c Pearson’s chi-square tests were conducted in two analytical steps: (i) by school size, comparing small schools (10–99 students) with not-small schools (≥100 students, combining medium and large schools); and (ii) within the subsample of small schools only, comparing SES terciles (low vs. medium vs. high).

d Counties in which schools were operated were grouped into three SES terciles (low, medium, high) based on composite economic indicators (*Economx, 2022*).

SES, socioeconomic status.

### Physical activity

Rhythmic movement was incorporated into daily physical education in 56.8% of schools overall (Table 3). Stress-relief techniques were adopted at comparable levels across groups (around 70%). Roughly one in eight schools reported using none of these approaches. We observed significantly lower access to after school sport in any forms in small schools than in medium and large schools (*P* < .001), though effect sizes were small. Free access to school facilities was reported by 71% of not-small schools, but only in 55.9% of small schools. Free or subsidized access to external facilities was available in just 28.1% of small schools. Free in-school sport activities were provided by 81.6% of not-small schools but only by 67.8% of small schools. Fee-based in-school sport programs were uncommon across all small schools (e.g. 5.2% in low SES and 8.9% in high SES small schools). No after-school sport access of any form was reported by 10.3% of small schools overall.

### Prevention programs targeting obesity-related issues

Programs targeting internet and digital addictions were present in 62% of medium and large schools, but only in 53.1% of small schools (*P* = .003, *V* = 0.06) (Table 3). Body image perception disorder prevention was the least common overall (21.1%), and even lower in low SES small schools (13.0%). Programs addressing bullying and school violence were implemented in 51.1% of schools overall, but in just 37.2% of small schools (*P* < .001, *V* = 0.12).

### School support to parents

The median agreement score on the statement that the school supports parental health literacy and healthy lifestyle choices was 6.0 (95% CI: 6.0–6.0) across all schools, 5.5 (95% CI: 5.0–6.0) in small schools, 5.0 (95% CI: 5.0–6.0) both in low SES and high SES small schools.

### Implementation across HPS global standards

To provide a more integrated view of implementation, aggregated indicators aligned with the HPS Global Standards were constructed (Table 4), revealing a clear gradient across domains. Overall, curriculum-related components showed the highest level of implementation (71.9%), followed by policy-related actions (61.7%) and elements of the social-emotional environment (51.9%). In contrast, substantially lower levels were observed for the physical environment (39.7%), involvement of school health services (24.5%), and community partnerships (15.1%). Governance and leadership showed the lowest level of implementation across all domains (8.5%). Differences by school size were consistent with item-level findings. Small schools demonstrated slightly higher implementation in nutrition policies related actions, but showed systematically lower implementation in governance, physical environment, and health service involvement. Within the subsample of small schools, socioeconomic differences were most apparent in policy-related and community partnerships domains.

**Table 4.** Exploratory implementation indicators by HPS Global Standards, by school size and socioeconomic context (*N *= 2883)^a^

	All schools (*N *= 2883)	Not-small schools (*N = *2563)	Small schools^b^ (*N *= 320)	Low SES small schools^c^ (*N *= 115)	High SES small schools^c^ (*N *= 101)
Standard 2—School policies and resources^d^	61.7	61.4	63.4	67.0	56.9
Standard 3—School governance and leadership^e^	8.5	8.9	5.4	5.6	4.7
Standard 4—School and community partnerships^f^	15.1	15.0	15.5	17.7	12.9
Standard 5—School curriculum^g^	71.9	72.3	68.6	70.9	68.3
Standard 6—School social-emotional environment^h^	51.9	52.6	46.2	43.3	48.1
Standard 7—School physical environment^i^	39.7	40.5	34.0	31.7	33.3
Standard 8—School health services^j^	24.5	25.0	19.8	20.4	18.6

a The mapping of questionnaire items to HPS Global Standards follows the conceptual framework presented in Table 1. Data are presented as the proportion (%) of implemented items within each standard, calculated at the school level and averaged across groups (i.e. 100% means that all schools implemented all items within a given standard). Indicators are descriptive summary measures derived from multiple items; no statistical tests were performed. Standard 1 (government policies and resources) was not assessed, as the survey focused on school-level implementation.

b Cut-off points (10–99 students) were selected in accordance with the official thresholds used in the Hungarian public education indicator system (*Hungarian Academy of Sciences, 2017*).

c To account for socioeconomic context, counties in which schools were operated were grouped into three SES terciles (low, medium, high) based on composite economic indicators (*Economx, 2022*).

d Based on Q10–Q11 (participation in School Milk and Fruit and Vegetable Schemes); Q12-1 (compliance with Public Catering Act); Q12-7 (ensuring special dietary needs).

e Based on Q5 (health promotion working group); Q6-1–6 (involvement of teachers, school doctor, nurse, psychologist, parents, students in HP program development).

f Based on Q6-5 (parental involvement); Q12-6 (use of local products in public catering); Q30 (programs to increase parental health literacy and healthy lifestyle choices).

g Based on Q13-1 (nutrition education); Q13-7 (conscious shopping education).

h Based on Q21-1 (Rhythmic movements to music, e.g. dance); Q21-2 (stress-relief techniques); Q24-5 (digital addiction prevention); Q24-6 (prevention of body image perception disorders); Q24-7 (bullying prevention).

i Based on Q13-2 (healthy menu in public catering); Q13-4 (water fountains); Q13-5 (school garden); Q13-6 (educational kitchen); Q22-1–4 (access to sports facilities and activities).

j Based on Q6-2–4 (school doctor, nurse, psychologist involvement); Q13-8 (school health day).

## Discussion

This study provides a comprehensive assessment on the implementation of school health promotion in Hungary and identifies substantial gaps overall, as well as disparities related to school size and socioeconomic context. The findings indicate that although a legal framework for HHP is in place, its translation into everyday school practice remains limited in several areas.

First, at school level, governance structures appear weak, and only a small proportion of schools reported having a dedicated health promotion working group. Within the HPS framework, such structures are considered central for showing the commitment of school leadership, and ensure that the necessary human and financial resources are in place [31]. Second, participatory planning remains limited. Although the whole-school approach emphasizes the involvement of students, parents, and health professionals, our results suggest that decision-making is still largely narrowed, with very low stakeholder engagement. This reduces shared ownership as well as local and school community engagement. Given these findings, schools appear to require targeted structural support to strengthen governance capacity. National or regional authorities could consider simplified governance models, such as shared inter-school coordination platforms, regional health promotion facilitators, or externally supported planning templates. Such approaches would align with the governance and leadership component of the HPS framework while acknowledging the overstretched workload of school staff [5].

Third, cooperation with local food producers in school catering was uncommon, despite its potential to support healthier nutrition, environmental goals and local economies. This signals missed opportunities for mutual benefits. Fourth, elements of an enabling physical environment were not widely available, as relatively few schools reported having water fountains or school gardens. While these require investment, they represent opportunities for practicing skills and knowledge necessary to support healthy lifestyles. Finally, prevention programs targeting body image and perception disorders were among the least frequently reported activities. Given the rising concern about body image issues in children and adolescents [2], this gap may indicate that schools prioritize more traditional health topics while emerging psychosocial risks receive less attention.

At the same time, several findings point to areas where school health promotion appears to be functioning well. Participation in the national Fruit and Vegetable Scheme was widespread, and most schools reported compliance with the public catering regulation, suggesting that centrally regulated nutrition policies can achieve broad reach. Similarly, nutrition education was reported by nearly all schools, indicating that health-related content is well embedded in the educational curriculum. These findings align with evidence that curriculum-based and policy-supported interventions are among the most consistently implemented components of the HPS approach [8].

Positive patterns were also observed in the physical activity domain. Many schools integrated stress-reduction techniques into daily physical education, and the availability of free in-school sport activities was high, both of which support regular physical activity and mental well-being. In addition, prevention programs addressing digital addiction were relatively common, supporting growing awareness of emerging health risks in the school-age population.

In line with the item-level assessment, the standard-level analysis reinforced the structural imbalance in implementation, where curriculum-embedded and centrally regulated elements are well established, while domains requiring local coordination, resources, and cross-sector collaboration remain underdeveloped.

Regarding school size, clear differences emerged highlighting both relative strengths and structural vulnerabilities of small institutions. Participation in national nutrition schemes was higher among small schools, especially those operating in lower SES areas. This suggests that centrally organized, fully standardized schemes with predefined logistics and funding mechanisms may function as important equalizers. Similarly, teachers in small schools more frequently reported acting as role models in promoting healthy eating, which may reflect the more intimate social environment and closer interpersonal relationships typical of smaller school communities [17]. However, it is important to note that these differences were generally associated with small effect sizes (Cramér’s *V* mostly < 0.10, with only a few values around 0.12–0.13), indicating that, although statistically detectable, the magnitude of size-related differences was modest.

These strengths coexist with notable structural weaknesses. Governance indicators were consistently poorer among small schools, as reflected by the lower presence of dedicated working groups and more limited stakeholder involvement in planning. Furthermore, small schools were less likely to report ensuring public catering for students with special dietary needs, despite the fact that the Public Catering Act has mandated it since 2014. This gap may reflect logistical and financial constraints, as smaller institutions often depend on external catering services and have limited capacity to ensure individualized provision.

Patterns in after-school physical activity provision further demonstrate systemic disadvantages for small schools. Most concerning, over one in ten small schools reported no extracurricular sport access at all. While the physical education (PE) curriculum is commonly recognized as the major vehicle for increasing physical activity in schools, physical activity recommendations for children and adolescents cannot be met through PE alone, and extra-curricular sport has a key role to fulfill these guidelines [32]. Partnerships with local municipalities, sports clubs, or neighboring schools may help compensate for restricted on-site facilities. Policy instruments that incentivize inter-institutional cooperation could strengthen the physical and social environment domain of health promotion without placing disproportionate burdens on small institutions. In addition, prevention programs targeting digital addictions and bullying were less frequently implemented in small schools. While smaller school environments are often perceived as safer and more cohesive, these findings indicate that smaller size does not automatically translate into stronger preventive engagement.

Within the subsample of small schools, several differences emerged across SES contexts. Although based on small absolute numbers, we observed a tendency toward greater parental involvement in health promotion planning in low SES small schools. Given the limited case numbers, this finding should be interpreted cautiously; however, it may indicate stronger reliance on community engagement and informal participation mechanisms in socioeconomically constrained settings. Tight cooperation between schools and communities is a well-documented feature of rural areas. For instance, the PISA 2015 report found that parents in rural settings tend to be more engaged with schools than those in urban areas [33]. Their involvement often extends beyond formal roles, including contributions to fundraising, building maintenance, extracurricular programs, and even direct teaching support.

More pronounced differences were observed in the implementation of nutrition-related policies. Participation in the School Milk Scheme was substantially higher among low SES small schools than among high SES small schools. This pattern suggests that centrally funded and standardized national programs play a particularly important compensatory role in disadvantaged areas, thereby supporting the equity-oriented objectives within the whole-school approach. However, reliance on these schemes may also reflect greater baseline food insecurity.

Elements of the physical and learning environment also varied by SES. School gardens and educational kitchens were more frequently reported in high SES small schools, reinforcing existing inequalities in experimental learning opportunities [34]. As these elements contribute to practice-based learning and the creation of a health-supportive school environment, their uneven distribution may reflect broader structural inequalities affecting the implementation of the whole-school model. This may require earmarked financial or technical support in disadvantaged areas. At the same time, low SES small schools reported greater use of local products in menu planning, which may indicate closer integration with local supply networks and community-level economic structures. Finally, despite the known correlation between the prevalence of overweight and obesity and low SES status [35], as well as between weight status and risk of bullying, poor body image, and sedentary behaviors [36, 37], low SES small schools appear under-equipped in delivering specific prevention programs targeting these interlinked issues. Only about half of low SES small schools implemented initiatives to address internet and digital addictions, which are known contributors to sedentary lifestyles and excessive screen time that are key behavioral risk factors for obesity and other chronic conditions [37]. Even fewer offered body image-related education, despite the higher risk for distorted self-perception and low self-esteem among children with obesity [36], particularly in low-resource environments where stigma may go unaddressed [38]. The data also indicate that the implementation of anti-bullying programs is limited in low SES small schools, with only about one-third reporting such initiatives, despite the established link between overweight status and bullying victimization [36].

Overall, these findings suggest that socioeconomic context is associated with somewhat more pronounced variation in implementation than school size, although differences remain moderate in magnitude.

This study has some limitations. First, although the target population was Hungarian elementary schools, the questionnaire did not include a separate item on school level; therefore, the analyses could not distinguish between primary-only, lower-secondary, or multi-level institutions. Consequently, school-level composition could not be examined as a potential source of variation in implementation patterns. Second, the survey did not capture information on who completed the questionnaire within the school (e.g. principal, delegated staff member, or a collaborative team), which introduces the possibility of reporting bias and variability in how accurately implementation practices were represented. Third, most variables were measured using dichotomous items (i.e. presence or absence of certain HHP elements), which restricts the ability to capture depth, quality or intensity of implementation. The absence of implementation fidelity indicators prevents conclusions about the quality of delivery or effectiveness of reported activities, limiting interpretation to the presence or absence of specific components. Finally, comparisons by school size involved unbalanced group sizes, with a substantially larger number of not-small schools compared to small schools which may influence the precision of estimates and should be considered when interpreting the findings.

Taken together, these findings reveal that small schools, especially those in low SES areas, are structurally disadvantaged in several key domains of health promotion. While national policies and programs provide a foundation, their benefits are not distributed evenly. Addressing these disparities will require not only strengthened monitoring and enforcement, but also targeted investment in the schools least able to comply on their own. Without additional support, the existing framework risks perpetuating inequalities rather than reducing them.

## Supplementary Material

ckag143_Supplementary_Data

## Data Availability

The datasets presented in this article were obtained from the Ministry of Human Capacities, Hungary. This authority disposes all rights upon providing access to the datasets. Requests to access the datasets should be directed to Z.R., zsuzsa.rakosy@aok.pte.hu. Key points This is the first nationwide study to analyze implementation of school health promotion policies in nearly 3000 Hungarian schools, providing unprecedented insight into local practices. Despite a national mandate, substantial variation in implementation was observed, indicating that policy adoption does not guarantee effective delivery at school level. Centrally coordinated, standardized programs showed more consistent implementation than local initiatives, highlighting the importance of structural support mechanisms. Socioeconomic context was associated with somewhat greater variation in implementation than school size, although most differences were modest in magnitude. Small schools showed structural vulnerabilities, including weaker governance, limited access to health professionals, and fewer opportunities for extracurricular physical activity calling for targeted resource allocation.
