# Supplementary material for: Disparities in practice: implementation of health promotion policies in 2883 Hungarian schools with a focus on small-sized institutions
Source: Eur J Public Health. 2026 Aug 13;36(5):ckag143. doi: 10.1093/eurpub/ckag143 (PMC13472674; doi:10.1093/eurpub/ckag143)
Supplement: Supplementary file 1 [file ckag143_supplementary_data.docx]

**Figure 1.** County-level percentage distribution of elementary schools in 2019/2020 (1) across Hungary (*black*), (2) in the survey sample (*dark grey*) and (3) among small-sized schools (10-99 students) in the sample (*light grey*). Source: Hungarian Central Statistical Office (*28*)*.* Counties with low socioeconomic status (SES) are Nógrád, Szabolcs-Szatmár-Bereg, Békés, Somogy, Jász-Nagykun-Szolnok and Borsod-Abaúj-Zemplén; counties with high SES are: Budapest, Fejér, Győr-Moson-Sopron, Komárom-Esztergom, Pest, Vas and Veszprém (*30*).
